# “Bird Song Metronomics”: Isochronous Organization of Zebra Finch Song Rhythm

**DOI:** 10.3389/fnins.2016.00309

**Published:** 2016-07-06

**Authors:** Philipp Norton, Constance Scharff

**Affiliations:** AG Verhaltensbiologie, Freie Universität BerlinBerlin, Germany

**Keywords:** zebra finch, birdsong, rhythm, pulse, music, gestures

## Abstract

The human capacity for speech and vocal music depends on vocal imitation. Songbirds, in contrast to non-human primates, share this vocal production learning with humans. The process through which birds and humans learn many of their vocalizations as well as the underlying neural system exhibit a number of striking parallels and have been widely researched. In contrast, rhythm, a key feature of language, and music, has received surprisingly little attention in songbirds. Investigating temporal periodicity in bird song has the potential to inform the relationship between neural mechanisms and behavioral output and can also provide insight into the biology and evolution of musicality. Here we present a method to analyze birdsong for an underlying rhythmic regularity. Using the intervals from one note onset to the next as input, we found for each bird an isochronous sequence of time stamps, a “signal-derived pulse,” or pulse^S^, of which a subset aligned with all note onsets of the bird's song. Fourier analysis corroborated these results. To determine whether this finding was just a byproduct of the duration of notes and intervals typical for zebra finches but not dependent on the individual duration of elements and the sequence in which they are sung, we compared natural songs to models of artificial songs. Note onsets of natural song deviated from the pulse^S^ significantly less than those of artificial songs with randomized note and gap durations. Thus, male zebra finch song has the regularity required for a listener to extract a perceived pulse (pulse^P^), as yet untested. Strikingly, in our study, pulses^S^ that best fit note onsets often also coincided with the transitions between sub-note elements within complex notes, corresponding to neuromuscular gestures. Gesture durations often equaled one or more pulse^S^ periods. This suggests that gesture duration constitutes the basic element of the temporal hierarchy of zebra finch song rhythm, an interesting parallel to the hierarchically structured components of regular rhythms in human music.

## Introduction

Rhythm is a key element in the structure of music and can be defined as the “systematic patterning of sound in terms of timing, accent and grouping” (Patel, 2008, p. 96). These patterns can be either periodic (i.e., regularly repeating) or aperiodic. A special case of a periodic pattern is an isochronous one, where the time intervals between successive events share the same duration. In many types of music across the world, including the Western European (Patel, 2008, pp. 97–99) and African (Arom, 1991, p. 211) traditions, the timing of sonic events, mostly note onsets, is structured by a perceptually isochronous pulse (Nettl, 2001). This pulse is a cognitive construct that is usually implicit rather than being materialized in the acoustic signal itself (Arom, 1991, p. 230; Fitch, 2013). For the purpose of this article we will call this the “perceived pulse,” or pulse^P^. In all but the simplest of rhythms not all notes fall on the pulse and some pulses occur in the silence between notes. Therefore, the intervals between the notes in a piece are rarely isochronous, but many note onsets align to an isochronous pulse. In some musical styles, variations of tempo—and therefore pulse—are used for artistic effect (e.g., accelerando and ritardando in classical music), while in others the tempo remains constant throughout a piece or performance (e.g., Central African music; Arom, 1991, p. 20). Often the pulse is further organized by a metrical structure, the recurring hierarchical patterning of strongly, and weakly accented events. In a waltz, for example, the pulse is perceptually divided into groups of three, of which the first one—the so-called downbeat—is perceived as more strongly accented than the following two (“*one*, two, three, *one*, two, three”). In this example, pulses on the lower level of the metrical hierarchy, i.e., every pulse, happen at three times the tempo of the higher level, consisting of only the strong pulses. The process of finding the pulse and frequently the subsequent attribution of meter allow us to infer the beat of a piece of music.

If you have ever danced or clapped your hands along to music, you have already encountered one function of a regular pulse: it facilitates the coordination of synchronized movements through a process called “beat perception and synchronization.” It also provides musicians with a common temporal reference that is necessary for coordinated ensemble performance (Arom, 1991, p. 179; Patel, 2008, pp. 99–100). Furthermore, expectations and the interplay of successful anticipations and surprises emerging from these expectations are thought to drive the “emotive power” of human music (Huron, 2006). Pulse and meter, as well as deviations thereof, can build anticipations in the time-domain that subsequently are either fulfilled or violated.

How did such an apparently universal aspect of human music evolve? Several authors have stressed the importance of a cross-species comparative approach to gain insights into the evolution of music (Hulse and Page, 1988; Carterette and Kendall, 1999; Hauser and McDermott, 2003; Fitch, 2006; Patel and Demorest, 2013). Crucial to this endeavor is the realization that the music faculty hinges on a variety of interacting perceptual, cognitive, emotional, and motor mechanisms that may follow different evolutionary trajectories. It is therefore helpful to break down the music faculty into these different components and investigate which of them are present, either by homology or analogy, in non-human animals (Fitch, 2006, 2015; Ravignani et al., 2014; Honing et al., 2015).

One critical component is our capacity for vocal learning. It allowed us to develop speech as well as song, which is assumed to be universal to human music (Nettl, 2001; Trehub, 2001; Brown and Jordania, 2011). Of the many species that produce vocalizations or other acoustic signals of varying complexity, only a few are well known to rely on developmental learning to acquire some of their adult vocalizations, e.g., songbirds, hummingbirds, and parrots as well as several species of bats, some marine mammals, and elephants (rewieved by Petkov and Jarvis, 2012).

Birdsong in particular has caught the interest of researchers for its putative musical features (Kneutgen, 1969; Dobson and Lemon, 1977; Marler, 2001; Baptista and Keister, 2005; Taylor, 2013; Rothenberg et al., 2014). It has frequently inspired human music and prompted composers to incorporate it into their compositions (Baptista and Keister, 2005; Taylor, 2014). Birdsong and music might also share similar mechanisms and functions. For instance, the same regions of the mesolimbic reward pathway that respond to music in humans are active in female white-throated sparrows listening to conspecific song (Earp and Maney, 2012). Many bird species also coordinate their vocalizations by simultaneous or alternating chorusing (reviewed by Hall, 2009) or have been shown to temporally coordinate bodily movements in a dance-like manner with song during courtship (e.g., Prum, 1990; Patricelli et al., 2002; DuVal, 2007; Scholes, 2008; Dalziell et al., 2013; Ota et al., 2015; Soma and Garamszegi, 2015). Whether zebra finches (*Taeniopygia guttata*) coordinate singing among individuals has not been studied, but they do integrate song and dance during courtship in a non-random choreography (Williams, 2001; Ullrich et al., 2016). As in human ensemble music and dance, an isochronous pulse might serve as a temporal reference for duetting and dancing birds, facilitating the temporal coordination of vocalizations, and movements. A recent study by Benichov et al. (2016) showed that zebra finches are also adept at coordinating the timing of unlearned calls in antiphonal interactions with a robot producing isochronously spaced calls. When the robot produced some additional calls, timed to coincide with the bird's response, both males and females quickly adjusted their calls to avoid jamming, successfully predicting the regular call pattern of the robot. The forebrain motor pathway that drives learned song production in male zebra finches seems to play a major role in this precise and flexible temporal coordination, not only in males but also in females that do not sing and have a much more rudimentary song system (Benichov et al., 2016). The capacity for “beat perception and synchronization” that enables humans to extract the pulse from a complex auditory signal and move to it has so far been found only in several species of parrots (Patel et al., 2009; Schachner, 2010; Hasegawa et al., 2011) and a California sea lion (Cook et al., 2013). Since human music was used as a stimulus in these studies it is not clear how these findings relate to the animals' own vocalizations: is there regularity in any learned natural vocalization signal that permits extraction of a regular pulse?

Song production in zebra finches has been successfully used as a model for studying vocal learning and production for several decades, motivated by its parallels to speech acquisition at behavioral, neural, and genetic levels (reviewed by Doupe and Kuhl, 1999; Bolhuis et al., 2010; Berwick et al., 2012). Therefore, a large body of knowledge exists about zebra finch song structure and development as well as their neurobiological basis. Zebra finch song learning and production is controlled by a neural network of specialized song nuclei (Nottebohm et al., 1976; Bolhuis et al., 2010). The nucleus HVC, cortical in nature, significantly contributes to the coding of song. Different ensembles of neurons fire short, sparsely occurring bursts of action potentials which, through a series of downstream nuclei, translate into a motor code controlling particular ensembles of muscles of the vocal organ (Hahnloser et al., 2002; Fee et al., 2004; Okubo et al., 2015). The level of resolution of our knowledge about how behavior is neurally coded is much finer grained in songbirds than in humans. So, while the present study in songbirds is guided by what we know about rhythm from human music it has the potential to shape our inquiry into the neural basis of human rhythm production and perception. The highly stereotypic structure of zebra finch song and the fact that it remains largely unchanged in the adult bird contributes to making it a good target for first investigations of periodicity, compared to more complex singers. We therefore analyzed zebra finch song rhythm, asking whether an isochronous pulse can be derived from the timing of its notes (signal-derived pulse; pulse^S^).

## Materials and methods

### Birds

This study used 15 adult male zebra finches, aged between 384 and 1732 days at the time of song recording. They were bred and raised at the Freie Universität Berlin breeding facility. Before entering this study, they were housed together with conspecific males, either in a large aviary or in a cage sized 90 × 35 × 45 cm. In both cases they had acoustic and visual contact to female zebra finches held in other cages or aviaries in the same room. The rooms were kept under an artificial 12 h/12 h light/dark cycle at 25 ± 3⋅C. The birds had access to food, water, grit and cuttlebone *ad libitum* at all times. Birds in this study were solely used for song recording, a procedure for which the local authorities overseeing animal experimentation do not require a permit because it does not cause pain or discomfort. Information on the degree of relationship between the test subjects was only available for some of the birds. Of those, none were siblings, or had been raised by the same parents (3534, 4295, 4306, 4523, and g13r8). We cannot exclude dependencies in song structure arising from the possibility that pairs of birds were influenced by the same tutors.

### Recording

For song recording, each male was transferred into a separate cage (40 × 30 × 40 cm) inside a sound attenuation box (60 × 60 × 80 cm), kept under a 12 h/12 h light/dark cycle. Audio was recorded through cardioid microphones, mounted at about 2 cm distance from the center of the cage's front wall in each box. These were connected to a single PC through an external audio interface. Audacity 2.0.3 was used to record a single-channel audio track (WAVE file, 44.1 kHz, 16-bit) for each bird. Recording took place over a period of 3 years: 2013 (10 birds), 2014 (4) and 2015 (1) at varying times between 8 a.m. and 6 p.m. In addition to song recorded in isolation (“undirected song”) we also solicited so called “directed” song from 9 of the 15 males by exposing them to the sound and sight of a female finch in a transparent plastic box placed in front of the recording cage. Directed and undirected songs of the same bird were recorded within 1–3 days.

### Labeling

Recordings were segmented into smaller files of up to 10,000 s (2 h 46 min) length and for each bird the segment containing most song was used for the analyses. Then, an IIR Chebyshev high-pass filter with a 1 kHz cutoff was applied to remove low-frequency noise, using Avisoft Bioacoustics SASLab Pro 5.2.07 (henceforth SASLab). Note on- and offsets were determined by automatic amplitude threshold comparison in SASLab and saved as timestamps. All measurements obtained through this procedure were reviewed by visual examination of the song sonogram and corrected by hand where necessary. Timestamps of falsely identified elements (i.e., above-threshold noise) were removed. In the rare cases in which notes could not be reliably measured by hand (due to overlapping noise or recording artifacts), all timestamps from the entire song were discarded. Introductory notes as well as calls preceding or following song were measured but not included in the subsequent analysis. All remaining timestamps were exported to MathWorks MATLAB R2012b 8.0.0.783 (henceforth Matlab), which was used for the rest of the analysis.

**Figure 1:**
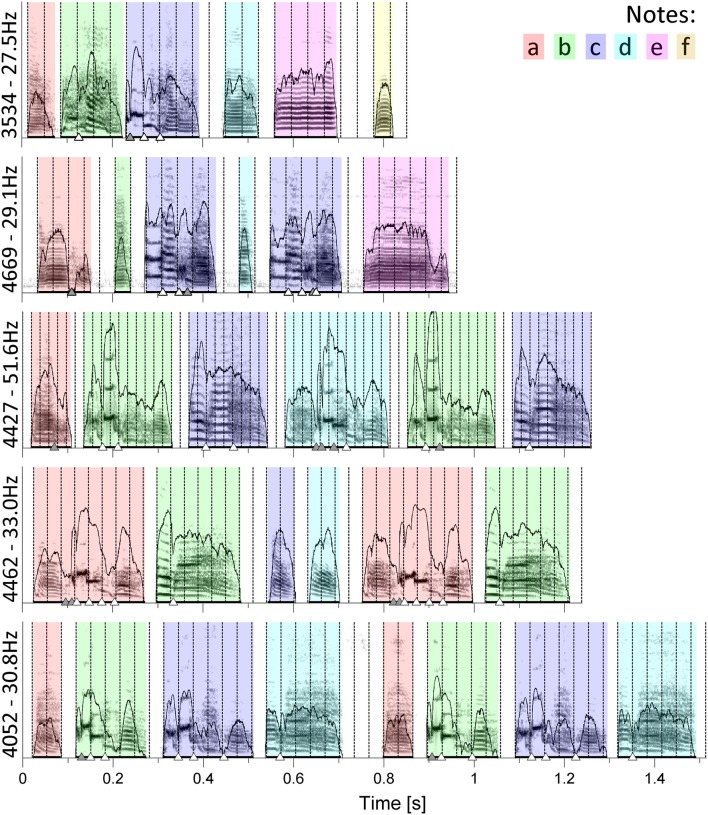
**Sonograms of song chunks from five different birds: 3534, 4669, 4427, 4462, and 4052 (top to bottom)**. For each song, note identity is indicated by color. Amplitude envelopes of the notes are outlined overlying the sonograms. Thicker black bars underneath the notes indicate note duration as determined by SASLab software. Isochronous pulses^S^ fitted to note onsets are marked as vertical dotted lines. Triangles indicate gesture transitions that either coincide with the pulse (white) or do not (gray, see Section Materials and Methods for details). Bird ID numbers and pulse frequencies are given to the left of each sonogram.

Song of zebra finches is composed of different notes, separated by silent intervals resulting from inhalation gaps. Notes consist of one or more sub-note elements, corresponding to neuromuscular gestures (hence called “gestures”; Amador et al., 2013). For the analysis, we labeled notes with alphabetical letters. A string of recurrent note sequences is called a motif. Slight changes of note order can result in motif variants. For each bird, notes with the same bioacoustic features within a motif were labeled with the same alphabetical letter (for examples see Figure 1). The number of different notes sung by each individual ranged from four to seven, labeled *a* through *g*. The most commonly sung motif received the note labels in alphabetical order. The interval between notes (hence called “gap”) following note *a* was labeled *a'*, following note *b b'* etc. Gaps were associated with the preceding syllable, as note duration correlates more strongly with the duration of the subsequent than the preceding gap (Glaze and Troyer, 2006). Introductory notes and calls were assigned different letters, and the corresponding timestamps were subsequently filtered out. When the first note of a motif was similar or identical to the introductory notes, it was considered the first note of the motif if it was present in each motif repetition and the gap between this note and the next was in the range typical of gaps within the motif. We used this criterion to distinguish between introductory notes and motif notes, because the former are separated by gaps of variable duration and the latter are not.

Rhythm analyses were performed on “chunks,” e.g., songs containing 1–10 continuously sung motifs. A new chunk started when a pause between two motifs lasted 300 ms or more. Chunks containing fewer than four notes (e.g., *abc*) or fewer than three bioacoustically distinct notes (e.g., *ababab*) were discarded in order to avoid “false positives,” e.g., finding a regular pulse^S^ as a mathematical consequence of few notes or low complexity. For each bird we analyzed between 12 and 68 undirected song chunks, consisting of 4–34 notes each (9.1 ± 4.5; mean ± std). Recordings of directed song contained 15–107 chunks, consisting of 4–42 notes (8.6 ± 5.9; mean ± std).

### Pulse matching

We used a generate-and-test (GAT) approach to find the pulse^S^ (signal-derived pulse) that best fitted the note onsets. Essentially, isochronous pulses, i.e., strings of timestamps of equal intervals, were created for a range of different frequencies. To assess the goodness of fit of each of those pulses to a particular recorded song, the root-mean-square deviation (RMSD) of all notes in the song chunk from their nearest single pulse (i.e., timestamp) was calculated. Specifically, we aimed to determine the slowest regular pulse that could coincide with all note onsets of the particular song under investigation.

To numerically determine the lower range of pulse intervals we therefore used the shortest measured inter-onset interval (IOI) for each tested song chunk and added 10% to account for variability. Lower frequency limits calculated this way ranged from 5.5 Hz (bird 4042) to 14.9 Hz (bird 4669). Starting there, the pulse frequency was incremented in 0.01 Hz steps up to 100 Hz. Preliminary investigation revealed that the best fitting pulses very rarely had frequencies above 100 Hz.

For each chunk, the pulse that fitted note onsets best was determined in the following way. Each of the pulses of incrementing frequency (by 0.01 Hz steps) was displaced from the beginning of the recording by offsets ranging from zero to one period in 1 ms steps. For each offset of each pulse the RMSD was calculated. The offset at which the RMSD was minimal was regarded as the “optimal offset.” The result of this process was a list of pulses of different frequencies (e.g., 5.50, 5.51, …, 100 Hz) for each chunk and their respective minimal RMSD.

Because pulse frequency is mathematically related to RMSD, e.g., faster pulses are associated with lower RMSDs, we normalized the RMSD by multiplication with the pulse frequency, resulting in the “frequency-normalized RMSD” (FRMSD). The FRMSD, unlike the RMSD, does not exhibit this long-term frequency-dependent decrease (Supplementary Figure 1). The RMSD on its own is an absolute measure of deviation. In contrast, the FRMSD was used in this study, measuring the deviation relative to pulse frequency. Essentially it indicates how well the pulse fits, taken into account its tempo. We selected the pulse with the lowest FRMSD as the best fitting pulse for each chunk.

### Fourier analysis

A Fourier analysis was performed to confirm the results of the GAT pulse matching method (Saar and Mitra, 2008). To this end the note onset timestamps of each song were used to generate a point process, i.e., a number string with a 1 ms time resolution, which was 1 at note onsets and 0 elsewhere. After performing a fast Fourier transform (FFT) on this string, we took the frequency of maximum power for each chunk (within the same Hz limits as above) and compared it to the frequency given by the GAT method.

### Gesture transitions

Examination of the sonograms showed that not only note onsets, to which the pulses were fitted, but also onsets of distinct bioacoustic features within notes, corresponding to neuromuscular gestures, coincided with the pulse remarkably often. We identified possible time points of these gesture transitions quantitatively through a previously published algorithm that determines significant local minima in the amplitude envelope (Boari et al., 2015). Amplitude minima occur not only on gesture transitions, but also within gestures and notes of quasi-constant frequency (e.g., note *e* of 3534, Figure 1). Thus, we selected from the time points produced by the algorithm only those as gesture transitions that corresponded to clear discontinuities in the frequency trace, identified by visual examination of the sonograms. The percentage of gesture transitions that fell within certain ranges around the pulse, namely one tenth, one sixth, and a quarter of the pulse period, were calculated. In Figures 1 and **3** gestures with a distance of less than one sixth of the pulse period to the nearest pulse are highlighted.

### Clustering

Visual examination revealed that the frequencies of the best fitting pulses of all song chunks from each bird tended to form clusters with individual values scattered between clusters (Supplementary Figure 2). To quantify this impression we used agglomerative hierarchical clustering in ten birds, taking the group average of frequency distances as a dissimilarity measure. The dissimilarity threshold was set at 0.025 for all datasets. There was a significant positive correlation between cluster frequency mean and standard deviation (Linear regression; *R*^2^ = 0.21; *p* < 0.001; *n* = 78), i.e., pulse frequency clusters were more tightly packed, the lower their frequency and vice versa. In order to obtain comparable clusters, different frequency transformations (square root, log_e_, and log_10_) were applied pre-clustering and their effect on this correlation was tested. Clustering in this study was done on the basis of log_10_-transformed frequency data because log_10_-transformation led to clusters with the least frequency-dependent standard deviation (Linear regression; *R*^2^ = 0.0007; *p* = 0.824; *n* = 77).

### Modeling

To address whether the pulse frequencies found through the GAT method could also be detected with similar goodness of fit in any arbitrary sequence of notes, we developed two sparse models of song with varying degrees of randomization. These models produce sequences of timestamps comparable to the ones obtained from the song recordings and consist of on- and offsets of virtual “notes.” The pulse deviation of the recorded bird songs was then compared to that of these artificial songs. We used the results to test the hypothesis that note onsets in zebra finch song align to an isochronous pulse more closely than expected by chance.

The first model, called “random sequence” model (Model R), creates virtual notes and gaps of random duration, albeit within a certain range. It ignores the note sequence of the original song, instead picking a new duration for each individual note. Therefore, the note sequence is not consistent across motif repetitions (e.g., natural song *abcd abcd abcd* compared to artifical song *a'c'b'd' g'i'h'k' m'l'n'o'*). Model R creates a pseudorandom value for each individual note in the analyzed song chunk and uses that as the duration of the corresponding modeled note. These pseudorandom values are drawn from a Pearson distribution using Matlab's pearsrnd() function. The distribution's mean, standard deviation, skewness, and kurtosis are equal to the distribution of all observed note durations from either undirected or directed song, depending on which is to be modeled. The same is done for each gap, only this time the distribution is modeled on that of the observed gap durations. To be more conservative and avoid introducing high variability into the gaps of the model songs, outlier values and durations of gaps with unusually high mean and variability (gray points in **Figure 6**) were excluded in the creation of the pseudorandom number distribution.

Like model R, the second so called “consistent sequence” model (Model C) creates notes and gaps of random duration within the range of actually observed durations. Unlike model R though, model C takes the note sequence of the original song into account, keeping the duration of individual notes and their associated gaps in their sequence consistent across motif repetitions (e.g., natural song *abcd abcd abcd* compared to artifical song *a'b'c'd' a'b'c'd' a'b'c'd'*). In the first step of creating a virtual “song,” the different note types in the analyzed song chunk were determined (e.g., *a, b, c, d*). Then a set of 100 pseudorandom numbers were created for each note type of a bird, drawn from a standard normal distribution using Matlab's randn() function. These sets were then transformed to have their respective means equal a random value (drawn from a uniform distribution) between the minimum and maximum of the means of all durations of each observed note type. The standard deviation of all sets equals the mean of the standard deviations of the durations of each note type in the database. The same was done for each gap, only this time using the standard deviations and range of means of the gap durations as the basis for the set transformation. Model C draws a random element from the appropriate set for each individual note in the analyzed song chunk and its associated gap, and uses that value as the duration of the corresponding modeled note/gap. The note/gap type durations in this model were kept consistent not only across motif repetitions within a chunk, but also across all analyzed chunks of a bird. This was achieved through seeding Matlab's random number generator (RNG) before the creation of the duration sets during the modeling of each song chunk. The same seed value was used for all chunks of a single bird and different seed values were used for different birds. The RNG was seeded again before drawing the individual note/gap durations from the sets. Here, each chunk from a bird was assigned a different seed value. As a result, each modeled chunk used the same set of 100 durations for each note/gap type, but different values from that set were selected each time.

The deviations of two songs from their best fitting pulses cannot be compared if those pulses strongly differ in frequency. Just as the RMSD depends on the pulse frequency (described above), so does the FRMSD, as it measures deviation relative to pulse frequency. We therefore repeated the pulse matching process for both the recorded songs and the artificial songs, this time restricting the matched pulses to a certain frequency range that was different for each bird and identical for all recorded and artificial songs of one bird. Since we wanted to test whether we can find equally well fitting pulses for the artificial songs as we did for the recorded songs, we chose the mean of the largest frequency cluster of each bird as the center of the range. Furthermore, the upper bound of the range was twice the frequency of the lower bound. This assured that for any frequency outside of this range, either one integer multiple or one integer fraction of that fell within the range. We then compared the FRMSD values of all recorded songs and their best fitting pulse in their frequency range to those of the artificial songs. To exclude the possibility of the models producing particularly periodic or aperiodic songs by chance, the artificial song creation and subsequent FRMSD comparison were repeated 50 times for each song.

### Statistics

To test the differences in pulse deviation between bird song and model song or between song contexts (directed and undirected song), a linear mixed effects analysis was performed (linear mixed model, LMM) using the statistical programming language R 3.0.2 (R Core Team, 2013) with the package lme4 (Bates et al., 2014). FRMSD was entered into the model as fixed effect. As FRMSD increases with the number of notes in a chunk (Supplementary Figure 3), the latter was used as a random intercept. *P*-values were obtained by likelihood ratio tests of the full model vs. a reduced model without the fixed effect (FRMSD). One sample *t*-tests were used to test whether the percentage of gesture transitions occurred in certain ranges around the pulses significantly more often than expected by chance.

## Results

For each of the 15 analyzed adult male zebra finches we found an isochronous pulse^S^ (signal-derived pulse) that coincided with all note onsets, using two independent analysis methods. For both, we used a continuous undirected song sample from each bird. The analyses were performed on segments, called “chunks” that contained notes not separated by more than 300 ms. Each analyzed chunk consisted of 1–10 motifs, composed of repeated unique notes, varying between 4 and 7 depending on the bird. Using a generate-and-test approach (GAT; see Section Materials and Methods) we identified for each chunk of a bird's recording a pulse^S^ that fitted best to the note onsets, i.e., had the lowest frequency-normalized root-mean-square deviation (FRMSD; Figure 1).

### Pulse frequencies

**Figure 2:**
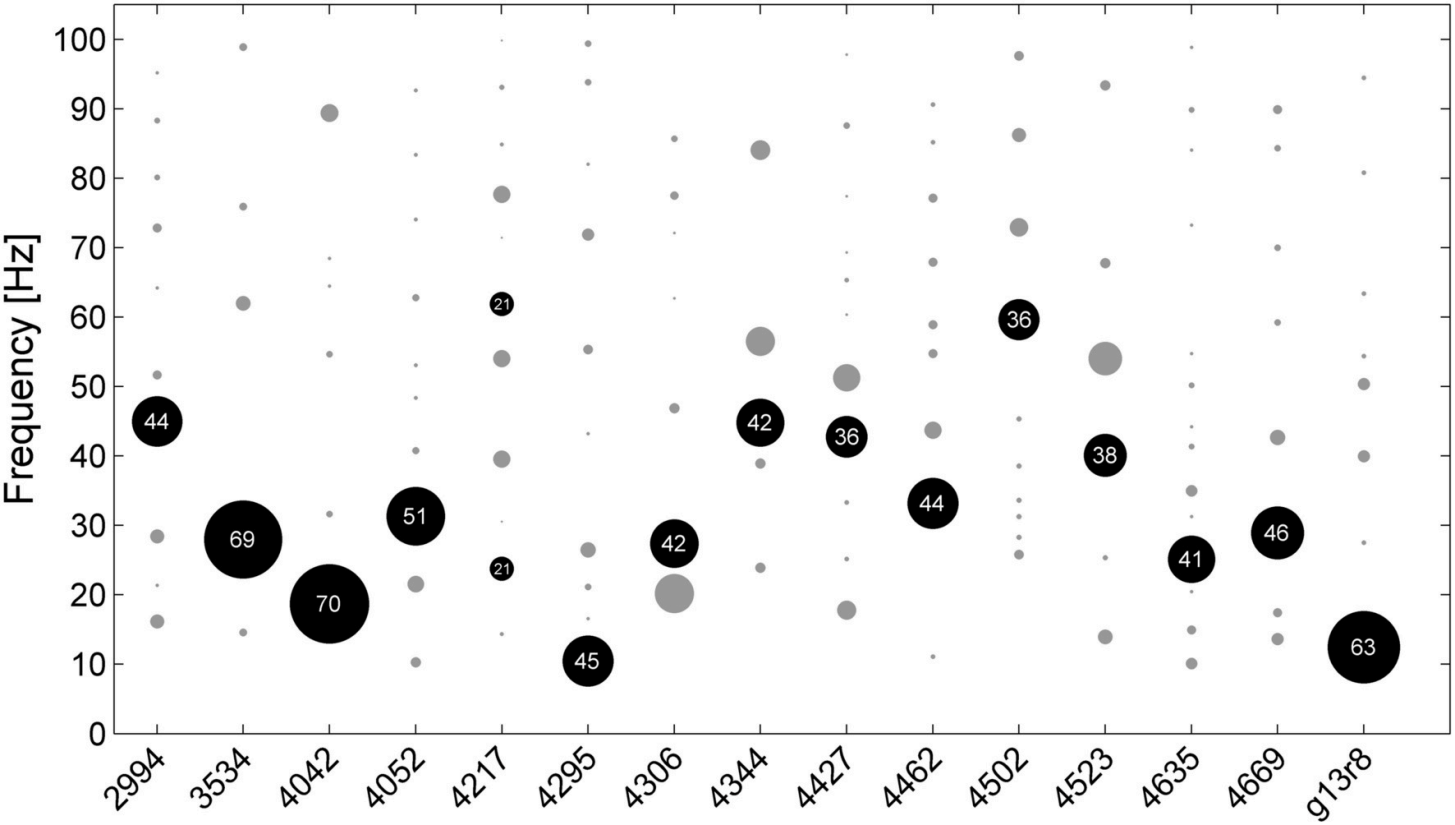
**Frequencies of the best fitting pulses^S^ for all analyzed chunks of undirected song for all 15 birds (bird ID numbers depicted on x-axis)**. Circles indicate frequency clusters as determined by hierarchical clustering analysis. Circle size corresponds to the percentage of chunks in the cluster relative to all chunks from the respective bird. The cluster containing the most chunks for each bird is black, the number inside the circle indicates the percentage of chunks within that cluster.

For all birds except one, a particular best fitting pulse dominated, e.g., between 36 and 70% of analyzed chunks from each bird clustered around a particular frequency (black circles in Figure 2). For 11 of 15 birds, best fitting pulse frequencies lay between 25 and 45 Hz. As a second analysis method to determine the best fitting pulses we applied a fast Fourier transform. We found that 91% of all chunks differed by < 0.25 Hz from the pulse frequencies identified by our GAT method.

In all birds a portion of songs were best fitted with pulses of different frequencies than those in the largest frequency cluster. Slight measurement inaccuracies may have lead to different pulses having a lower deviation than the putative “real” pulse in some songs. Song amplitude throughout the recordings varied slightly depending on the birds' position in the cage and the orientation of their heads during singing. This is likely to have introduced some variability in the note onset measurement by amplitude threshold detection. The use of a dynamic time-warping algorithm for onset detection should provide more accurate measurements (e.g., Glaze and Troyer, 2006). Another factor that might tie into the variability in pulse deviation is the fact that zebra finches gradually slow down by a small degree during bouts of continuous song (Glaze and Troyer, 2006).

### Gesture transitions

Often the best fitting pulse coincided not only with note onsets, but also with onsets of particular bioacoustic features within notes, corresponding to neuromuscular gestures. This was unexpected because the pulse was determined based on note onset times and not based on gesture transitions.

To quantify this observation, we identified possible time points of gesture transitions through an algorithm that determines significant local minima in the amplitude envelope (Boari et al., 2015). Out of these time points we selected those that coincided with clear discontinuities in the frequency domain of the song sonogram as gesture transitions. We did this for one song chunk from each of the 15 birds and found that overall 50.8% of gesture transitions fell within one sixth of the pulse period around single pulses (white triangles in Figure 1). If the gesture transitions were randomly distributed, 33.3% would be expected to fall in this range, as the range within one sixth of the period to either side of each pulse adds up to a third of total song duration. The percentage of gesture transitions that were within this range was significantly higher than the percentage expected by chance [one sample *t*-test, *t*_(14)_ = 2.894, *p* = 0.0118]. We found that the pulses also had a significantly higher coincidence with the gestures than expected by chance when we chose other ranges. Within one tenth of the period around pulses lay 34.3% of the transitions, significantly more than the 20% expected by chance [*t*_(14)_ = 2.315, *p* = 0.0363]. Within a quarter lay 65.9%, while 50% were expected if gesture transitions were randomly distributed [*t*_(14)_ = 2.639, *p* = 0.0195]. Inspection of the sonogram revealed many cases in which gesture duration equaled one or multiple pulse periods (for one pulse period see e.g., note *c* of bird 3534; *c* of 4669; *d* of 4427; *a* and *b* of 4462; *b* and *d* of 4052; for multiple pulse periods see *c* of 4427; Figure 1). In other cases multiple successive gestures added up to one pulse period (*c* of 3534; *c* of 4669; *b* of 4052). Note offsets did not systematically fall on the pulse, but in some cases notes consisting of a single gesture spanned one or more pulse periods (*d* of 3534; *b* and *e* of 4669; *c* of 4462; *a* of 4052). These observations imply a strong relationship between gesture durations and IOI.

**Figure 3:**
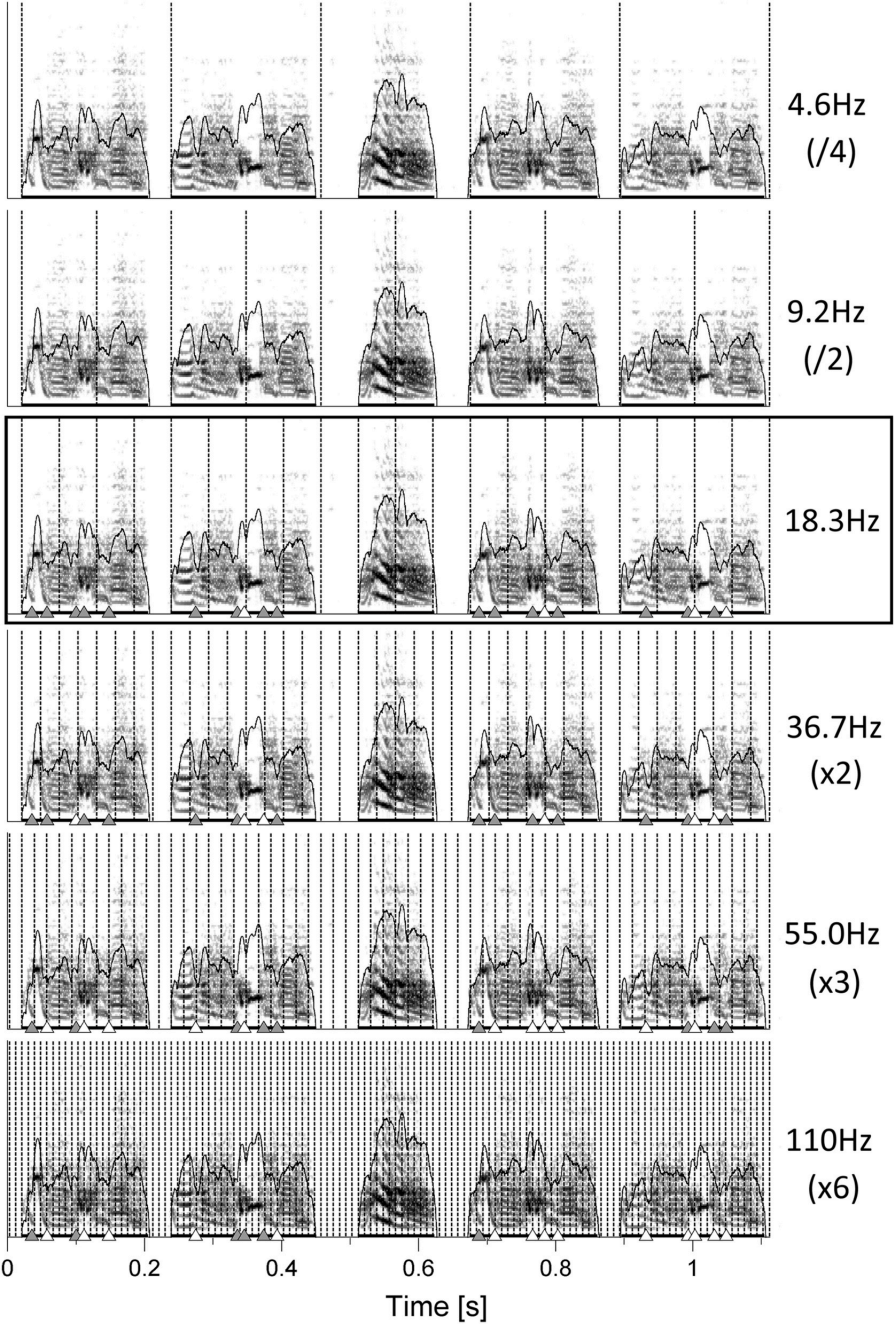
**Sonograms of one song chunk from bird 4042**. Pulses^S^ are the one best fitting on note onsets (18.3 Hz, black box) as well as integer fractions and multiples of that frequency. Triangles indicate gesture transitions that either coincide with the respective pulse (white) or do not (gray, see Section Materials and Methods for details).

Motivated by the unexpected finding that the pulses fitted not only note onsets but also many of the gestures, we wondered whether even shorter gestures would coincide with faster pulses, corresponding to integer multiples of the slowest fitting one. Interestingly, inspection of one bird under five additional pulse frequencies revealed increasingly higher coincidence of pulses with all observable gesture transitions (Figure 3).

### Directed song

Song directed by zebra finch males at females during courtship is less variable in various ways than when males sing so called “undirected” song (Sossinka and Böhner, 1980). During courtship, zebra finches deliver their song slightly faster than during undirected singing (Sossinka and Böhner, 1980; Cooper and Goller, 2006). In addition, notes and the sequence in which they are sung are produced in a more stereotyped manner from rendition to rendition during directed singing. Whether the duration of notes is also less variable in the directed than the undirected singing context is not known (Glaze and Troyer, 2006). To find out whether directed song had a faster pulse or whether the pulse fitted better due to lower variability (i.e., lower FRMSD) we recorded 9 of the previously analyzed birds also in a directed song context.

**Figure 4:**
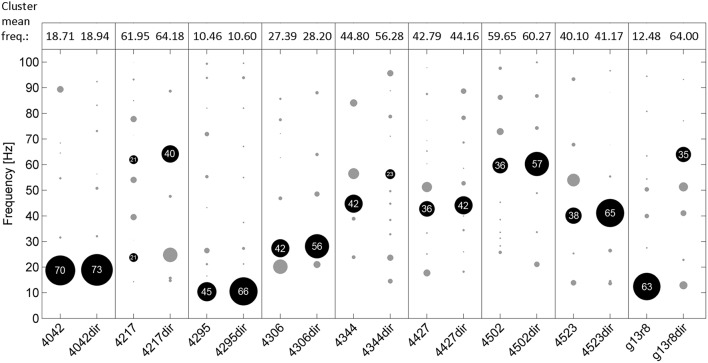
**Frequencies of the best fitting pulses^S^ for all analyzed chunks of undirected (left in each box) and directed song (right, e.g., “4042dir”) for 9 birds**. Circles indicate frequency clusters as determined by hierarchical clustering analysis. Circle size corresponds to the percentage of chunks in the cluster relative to all chunks from the respective bird and condition. The cluster containing the most chunks for each bird and condition is black and the percentage of chunks in that cluster is given. Mean frequency of those clusters is shown on top of the figure.

**Figure 5:**
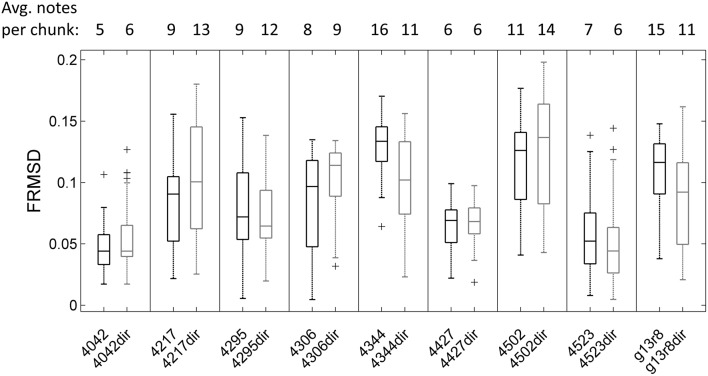
**Boxplot of frequency-normalized root-mean-square deviation (FRMSD) of note onsets to pulse^S^ for all undirected (left in each box) and directed song chunks (right in each box, e.g., “4042dir”) of 9 birds**. Outliers are marked by crosses. There was no significant difference in FRMSD between undirected and directed song (LMM; *p* > 0.05 for all 9 birds). Note that FRMSD increases with the number of notes in the chunk (Supplementary Figure 3), which was accounted for in the linear mixed model.

Mean pulse frequency of the largest cluster of undirected song was slightly lower than the nearest cluster in directed song in all birds (Figure 4). This is consistent with the fact that directed song is performed faster than undirected song (Sossinka and Böhner, 1980; Kao and Brainard, 2006; Woolley and Doupe, 2008), linked to a higher level of motivation during directed singing (Cooper and Goller, 2006). In 7 of 9 birds the pulse frequency best fitting most chunks was in the same range for undirected and directed songs. Interestingly, there was no significant difference in FRMSD between directed and undirected song (LMM; *p* > 0.05 for all 9 birds; Figure 5), indicating that note onsets in directed song do not appear to have a stronger or weaker periodicity than those of undirected song.

### Comparison to randomized model “song”

To evaluate the fit of note onsets to the pulses, we created artificial “songs” consisting of randomized note and gap durations and compared the deviations of their note onsets from an isochronous pulse to those of the recorded birds.

**Figure 6:**
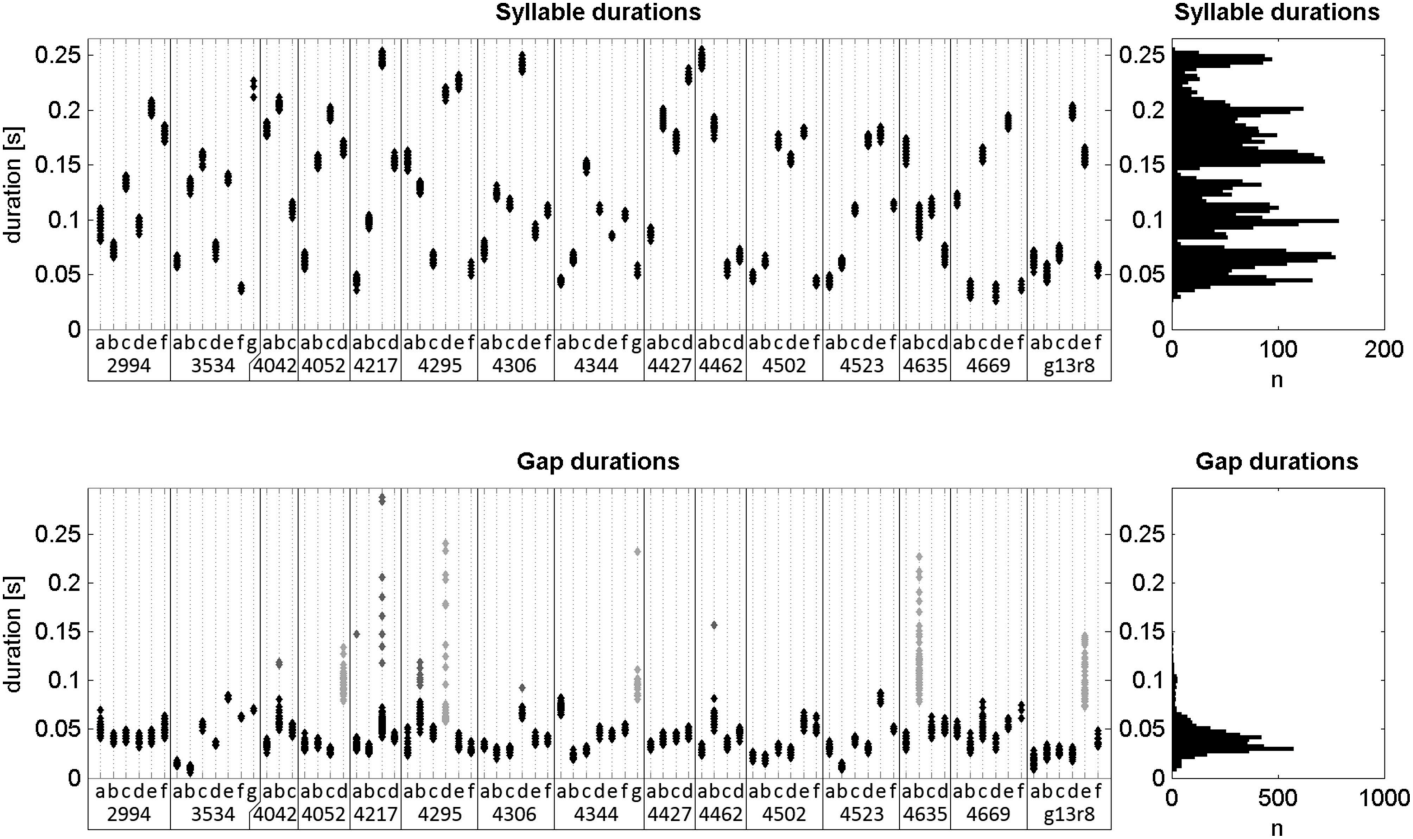
**Durations of all labeled notes and silent gaps of undirected song**. Left: Scatterplots of durations separated by note type and bird ID (x-axis). Gaps are categorized by the preceding note. Right: Histograms of all note and gap durations. The durations of all 5006 notes ranged from 26 to 256 ms (134 ± 60 ms; mean ± std). Of all gaps, 4456 were within song chunks and had durations between 6 and 288 ms (43 ± 23 ms; mean ± std). Of those, 305 were either considered outliers or were part of gap types that had a high mean duration and large variance (dark gray and light gray points, respectively). Excluding those, the remaining gap durations ranged from 6 to 88 ms (38 ± 12 ms; mean ± std).

The songs of the first model (“random sequence,” model R) do not replicate the note sequence of the recorded song. Instead a new pseudorandom duration is picked for each individual note and gap from a distribution modeled on that of the recorded notes and gaps. Through this comparison we could answer the question of whether a similar periodicity could be found in any arbitrary sequence of an equal number of (finch-like) song elements. We modeled the durations on the population of measured values of all birds in this study (Figure 6).

For each chunk we created 50 artificial songs with different randomized duration values each time and compared those to the recorded song chunks (see Section Materials and Methods for details). In overall 99% of comparisons bird songs had a lower pulse deviation (FRMSD) than the artificial songs created by model R (Table 1). In 88% of cases deviations were significantly lower compared to model songs, while the opposite never occurred (LMM; *p* < 0.05). The analyzed natural songs therefore match a regular pulse significantly better than expected by chance. In other words, all IOI of one bird are proportional to each other (i.e., integer multiples of the pulse period), unlike an arbitrary sequence of (finch-like) durations.

**Table 1:** **Results of the comparison between recorded undirected songs and model songs for all 15 birds**.

**Bird**	**Model R (random sequence)**				**Model C (consistent sequence)**			
	**Deviation**	***p* < 0.05**	**Deviation**	***p* < 0.05**	**Deviation**	***p* < 0.05**	**Deviation**	***p* < 0.05**
	**bird < model**		**model < bird**		**bird < model**		**model < bird**	
2994	100	100	0	0	95	73	5	0
3534	98	70	2	0	98	50	2	0
4042	100	100	0	0	93	82	7	0
4052	100	100	0	0	84	66	16	0
4217	100	100	0	0	100	98	0	0
4295	100	100	0	0	77	57	23	11
4306	100	100	0	0	98	86	2	2
4344	100	60	0	0	95	50	5	0
4427	100	100	0	0	82	50	18	2
4462	100	100	0	0	77	43	23	7
4502	84	0	16	0	59	5	41	0
4523	100	98	0	0	98	77	2	0
4635	100	100	0	0	66	39	34	14
4669	100	94	0	0	70	39	30	9
g13r8	100	100	0	0	18	9	82	70
Mean:	99	88	1	0	81	55	19	8

*Artificial song creation was repeated 50 times for each song chunk with different pseudorandom values for each repetition. Values are the percent of repetitions in which the pulse^S^ deviation (FRMSD) was lower in natural songs compared to model songs and vice versa (first and third column in each block) and the percentage in which this difference was statistically significant (LMM; p < 0.05; second and fourth column in each block). Column mean is given at the bottom*.

In most cases IOIs within one chunk are not completely independent of each other, as notes or whole motifs are repeated, and repetitions of notes and associated gaps are mostly very similar in duration. Thus, we compared the recorded songs to a second model (“consistent sequence,” model C), that preserves the sequence of the recorded song. In all artificial songs produced by model C for one bird, for example, the notes based on note *a* have a similar duration. In 81% of comparisons, FRMSD was lower in the natural song than in the model C songs (Table 1). It was significantly lower in bird songs in 55% and significantly lower in model songs in 8% of comparisons (LMM; *p* < 0.05). Model C songs performed better than model R songs in terms of pulse deviation, but still worse than the natural songs in the majority of cases. This leads us to conclude that the pulse is a result of the durations of the song elements as well as their sequence.

## Discussion

We showed here for the first time that the song of a passerine songbird, the zebra finch, can be fitted to an isochronous pulse^S^ (signal-derived pulse). Note onsets coincided with pulses of frequencies between 10 and 60 Hz (25–45 Hz for most birds) and at different frequencies for each individual. In female-directed song this periodicity was not significantly different from undirected song. In addition to note onsets, many of the transitions between gestures within complex notes coincided with the same pulse as well, more so than expected by chance. Finding a pulse in zebra finch song raises questions about the underlying neural mechanism and its behavioral function. We cannot offer definite answers but some suggestions:

Song is coded in HVC neurons projecting to nucleus RA (HVC_RA_) of the motor pathway. Different ensembles of those neurons fire at particular positions of each rendition of a song motif in a single, roughly 10 ms long, burst of action potentials (Hahnloser et al., 2002). Finding no connection between temporal firing of these neurons and note on- and offsets led to a working hypothesis, according to which HVC_RA_ neurons act together like a clock, producing a continuous string of ticks (“synfire chain”) throughout song on a 5–10 ms timescale (Fee et al., 2004). Additional evidence for a clock-like signal in HVC controlling song production comes from experiments in which HVC was locally cooled (Long and Fee, 2008). This caused song to slow down up to 45% across all timescales, including gaps, while only slightly altering the acoustic structure. Since neural activity in RA gives rise to the motor code for song production (Mooney, 2009), one could expect to see the periodicity of the synfire chain reflected in the temporal structure of song. The frequency of this periodic activity would be in the range of 100–200 Hz. The best fitting pulses found in this study, however, are between 3 and 10 times slower. This suggests that the timing of song notes is organized on a slower timescale, occurring only at every *n*th clock tick, with *n* depending on the individual. Since we found these slower pulses in the songs of all birds and the songs were made up of several different notes, we propose that additional mechanisms must operate to orchestrate the timing signals of the internal clock into higher hierarchical levels giving rise to the slower pulse.

One such mechanism was proposed by Trevisan et al. (2006) to explain the diverse temporal patterns in the songs of canaries (*Serinus canaria*). They constructed a simple nonlinear model of respiratory control that could reproduce the air sac pressure patterns recorded during singing. This model, in which respiratory gestures emerge as different subharmonics of a periodic forcing signal, could predict the effects of local cooling of canary HVC on song notes (Goldin et al., 2013). As in zebra finches, canary song begins to slow linearly with falling temperature. At a certain point, however, notes begin to break into shorter elements, as forcing, and respiration lock into a different integer ratio (e.g., from 2:1 to 1:1). Such a model might explain how a minimal time scale—e.g., in the form of an HVC synfire chain—could drive the timing of zebra finch notes on a subharmonic frequency. Zebra finch songs include more complex notes, in which several gestures of different duration are strung together in a single expiratory pulse. Our observation that gesture transitions preferentially coincided with the pulse on the note level, suggests that a similar mechanism might be responsible for periodic activation of the syringeal membrane.

Another study that recorded from HVC_RA_ in zebra finches found that they fired preferentially at so called “gesture trajectory extrema.” These comprise gesture on- and offsets as well as extrema in physiological parameters of vocal motor control within gestures, namely air sac pressure and membrane tension of the syrinx (Amador et al., 2013). This suggests that gestures might be the basic units of song production and that their timing is coded early in the song-motor pathway. It cannot be ruled out in this scenario that a number of neurons continue to fire throughout the song, sustaining a clock-like functionality (Troyer, 2013). In fact, a very recent paper using a range of methods to correlate HVC ensemble neural activity with song finds that HVC projection neurons exhibit a temporal sequence that does not occur preferentially with note onsets or offsets, nor with gesture transitions (Picardo et al., 2016). Be that as it may, our results imply that gestures transitions, like note onsets, contribute to song regularity. On average around half of the gesture transitions coincided with the pulse fitted to note onsets, significantly more than expected if they were randomly distributed. Those that did not, often occurred at the boundaries of gestures shorter than the pulse period, and successive short gestures often added up to one or multiple periods. These observations imply a strong relationship between gesture duration and IOI, where gestures constitute the lowest level of the temporal hierarchy. Notes are on a higher level of this hierarchy, combining one or more gestures and the intervening inhalation gaps. In this sense the rhythmic structure in zebra finch song is reminiscent of the relationship between notes and phrases in metrical rhythms of human music.

What might be the behavioral function of the periodic organization of song? Temporal regularity in an auditory signal can facilitate the anticipation of events. In the wild, zebra finches live in large colonies that provide a very noisy environment. Females have to attend to the song of a single male against a backdrop of conspecific vocalizations as well as other sources of noise. Temporal predictability of an auditory signal has been shown to enhance auditory detection in humans (Lawrance et al., 2014), a phenomenon from which zebra finches could benefit as well. Humans are also thought to possess a form of periodic attention. When asked to judge the pitch difference of the last of an isochronous sequence of 10 tones of different pitches to the first, they were more successful when the last tone was on the beat than when it came slightly early or late (Jones et al., 2002). This supports the idea that accurate expectation (i.e., when a stimulus might occur) has a facilitating effect on attention, improving the ability to assess what the characteristics of the stimulus are (Seashore, 1938; Huron, 2006). The benefit of successful anticipation of events is that it allows the optimization of arousal levels and therefore the minimization of energy expenditure (Huron, 2006). When female zebra finches were given the choice between undirected and directed song from the same individual, they preferred to listen to the latter (Woolley and Doupe, 2008). In this study the strength of this preference was negatively correlated with the variability in fundamental frequency of multiple renditions of harmonic stacks (parts of notes with clear harmonic structure and little frequency-modulation, e.g., the first two gestures of note *c* in 4669's song; Figure 1). This suggests that females attend to the pitch at specific times in a male's song and show a preference for males that are able to consistently “hit the right note.” If that is the case, it would be advantageous for them to be able to anticipate the timing of these structures. Since these gestures seem to be periodically timed, females could benefit from a form of periodic attention. Instead of maintaining a constant high level of attention throughout the song or establishing a new set of expectations for each individual male, they could then simply adjust the “tempo” of their periodic attention to fit that of the singer. Females possess most of the nuclei of the song system, including HVC and RA, albeit much smaller. Until recently, the function of these nuclei was largely unknown, although in canaries HVC is implicated in song recognition and discrimination (Halle et al., 2002; Lynch et al., 2013). Benichov et al. (2016) showed that following disruption of the song system, the ability for precise, predictive timing of call coordination is greatly reduced in both males and females. It is therefore probable that females use some of the same structures that enable males to produce song with high temporal regularity, to either assess the quality of this regularity, or to use it for the anticipation of other song features.

Whether zebra finches perceive the apparent periodicity in song and if so, on what timescale, is still an open question that is crucial for our understanding of their function. A recent study showed that ZENK expression was found to be elevated in several auditory nuclei after exposure to arrhythmic song, where inter-note gaps were lengthened or shortened, compared to natural song (Lampen et al., 2014). The observed differences in neural response suggest that rhythm plays a role in auditory discrimination of songs. In another study, zebra finches learned to discriminate an isochronous from an irregular auditory stimulus (van der Aa et al., 2015). The birds did not generalize this discrimination well across tempo changes, suggesting that they discriminated based on differences in absolute time intervals rather than relative differences (i.e., equal intervals in the isochronous vs. variable intervals in the irregular stimulus). Subsequently, zebra finches were asked to discriminate regular from irregular beat patterns, consisting of strongly accented tones with either a regular or a varying number of interspersed weakly accented tones. Here, some of the individuals were sensitive to the global pattern of regularity, but in general seemed to be biased toward attending to local features (ten Cate et al., 2016). The stimuli used in these studies, a series of metronome-like tones, lack features present in natural song—like timbre, pitch, and amplitude modulation—which might be necessary for regularity detection, or for the birds to perceive it as a relevant signal. Further studies are needed to uncover whether zebra finches perceive a regular pulse^P^ in song.

The pulses^S^ fitted to song notes in the present study were faster by multiple factors than those humans preferentially perceive in musical rhythm. The latter are in a tempo range of around 500–700 ms, which translates to a pulse^P^ frequency of 1.5–2 Hz (Parncutt, 1994; van Noorden and Moelants, 1999). Zebra finches do possess a higher auditory temporal resolution than humans (Dooling et al., 2002). It is important to note, however, that pulses^S^ in the current study were fitted to all note onsets and represent the lowest level pulse in terms of note timing. In human music the perceived pulse^P^ is usually slower than this low level pulse with notes occurring between successive beats. If birds perceive a pulse^P^ in song, one could expect it to be on a longer timescale as well, e.g., integer multiples of the pulse^S^ period, where some but not all notes coincide with the pulse^S^ (see the top sonogram in Figure 3 for an example).

Looking into the development of song regularity during song learning, especially in isolated juveniles, might provide further insights into whether periodicity is a result of song culture or whether it is neurally “hard-wired.”

## Author contributions

PN recorded songs and analyzed data; CS and PN designed study, prepared figures, interpreted results, drafted, and revised manuscript.

## Funding

This research was funded by the BMBF project “Variable Töne” (FKZ: 01GQ0961) and the Excellence Cluster “Languages of Emotion” project “Do birds Tango?” (201).

### Conflict of interest statement

The authors declare that the research was conducted in the absence of any commercial or financial relationships that could be construed as a potential conflict of interest.
