# Supplementary material for: “Bird Song Metronomics”: Isochronous Organization of Zebra Finch Song Rhythm
Source: Front Neurosci. 2016 Jul 6;10:309. doi: 10.3389/fnins.2016.00309 (PMC4934119; doi:10.3389/fnins.2016.00309)
Supplement: Supplementary file 1 [file DataSheet1.docx]

# Supplementary figure captions

**Supplementary Figure 1** – Frequency-normalized root-mean-square deviation (FRMSD, top) and root-mean-square deviation (RMSD, bottom) for one song chunk from bird 4052 (shown in the bottom sonogram in **Figure 1**). Isochronous pulses^S^ for this chunk were created between 9.47 and 100Hz in 0.01Hz steps. RMSD of note onsets to nearest single pulse were calculated for each pulse frequency and multiplication with that frequency yielded FRMSD. The RMSD, unlike the FRMSD, decreases non-monotonically with increasing frequency. The pulse with the lowest FRMSD was selected as the best fitting pulse (30.82Hz in this case). Note that the pulse of double frequency – at 61.64Hz – has an RMSD equal to the 30.82Hz pulse but a higher FRMSD.

**Supplemental Figure 2** - Frequencies of the best fitting pulses^S^ for all analyzed chunks of undirected song for all 15 birds (bird ID numbers depicted on x-axis). Lines next to points indicate which points belong to one cluster and numbers indicate the number of chunks in that cluster. This figure is identical to **Figure 2** in the main article, except that it shows individual points.

**Supplementary Figure 3** – FRMSD versus number of notes for all analyzed song chunks.
