# Supplementary figures and images for: “Bird Song Metronomics”: Isochronous Organization of Zebra Finch Song Rhythm
Source: Front Neurosci. 2016 Jul 6;10:309. doi: 10.3389/fnins.2016.00309 (PMC4934119; doi:10.3389/fnins.2016.00309)

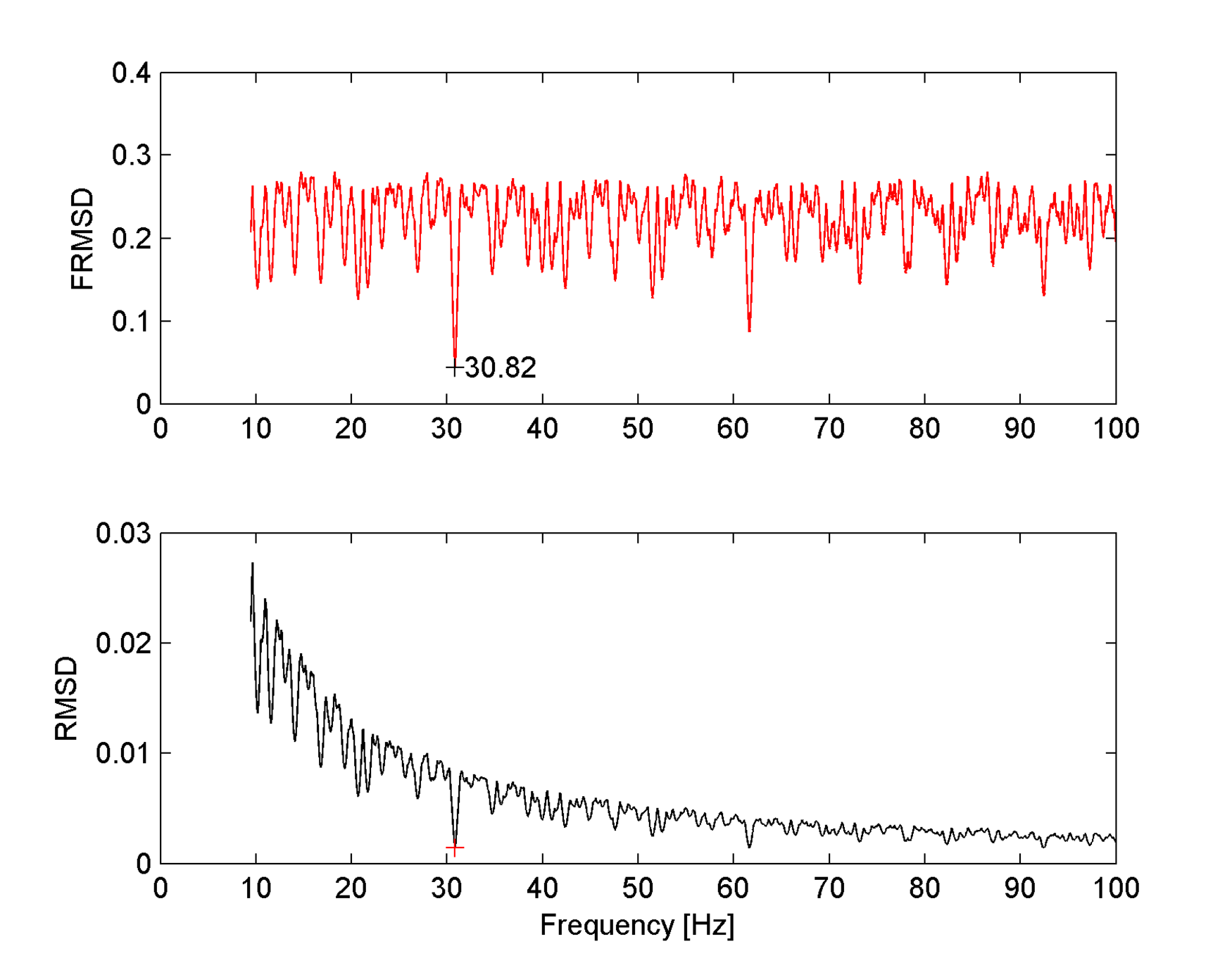

Supplement: Supplementary file 2 [file Image1.TIF]

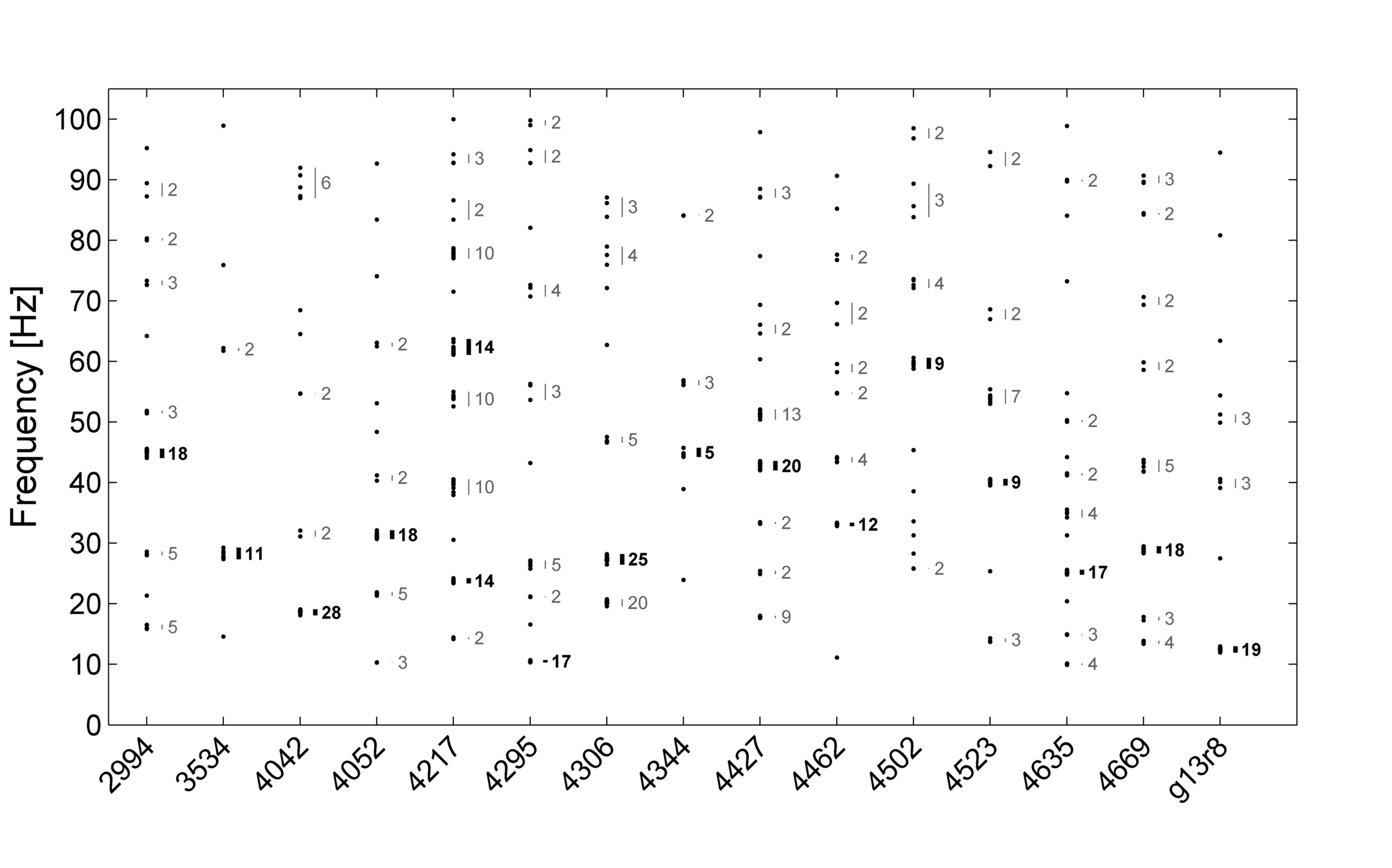

Supplement: Supplementary file 3 [file Image2.TIF]

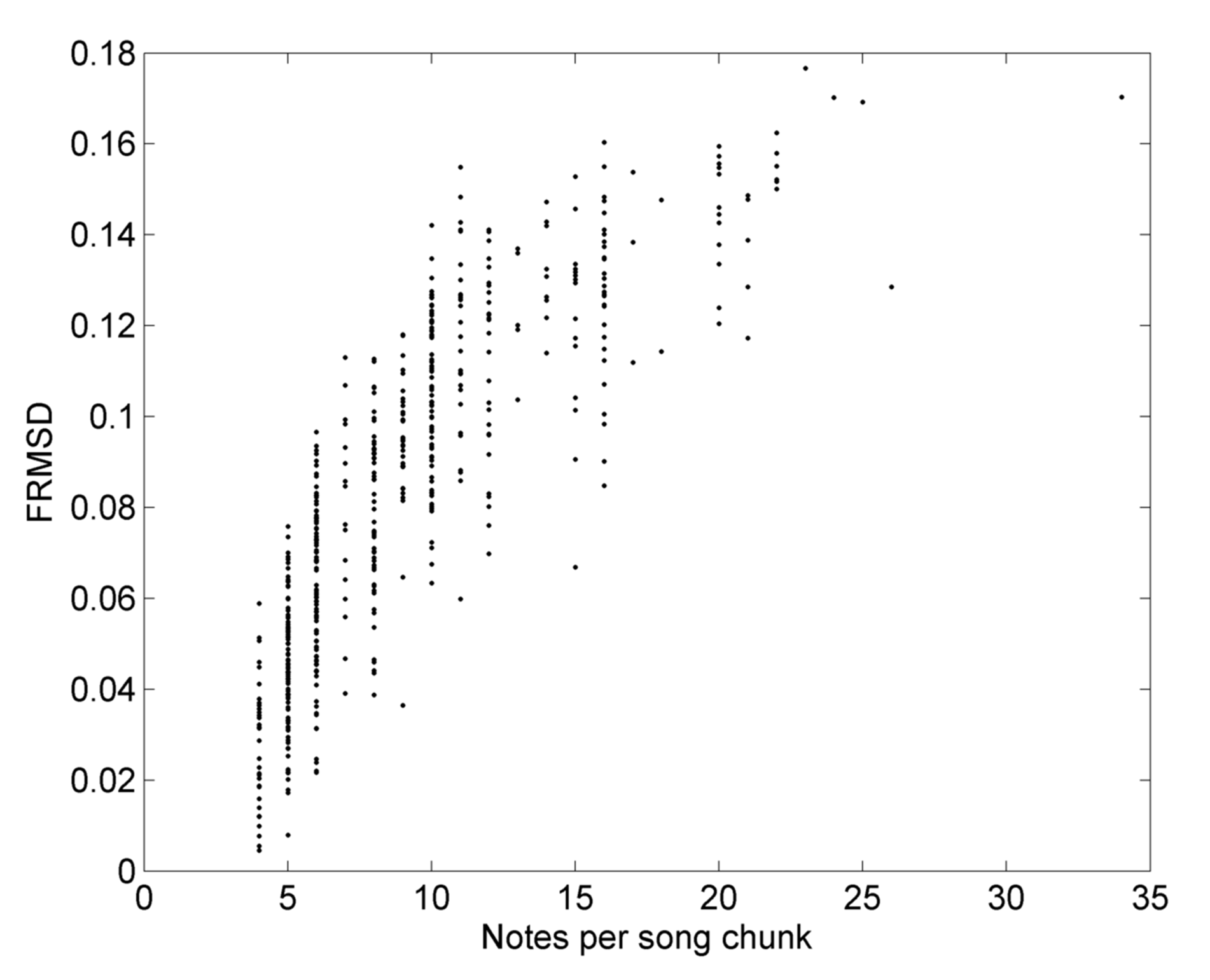

Supplement: Supplementary file 4 [file Image3.TIF]
